# Predicting Adolescent Idiopathic Scoliosis among Chinese Children and Adolescents

**DOI:** 10.1155/2020/1784360

**Published:** 2020-07-19

**Authors:** Bin Yan, Xinhai Lu, Qihua Qiu, Guohui Nie, Yeen Huang

**Affiliations:** ^1^Department of spine surgery, The First Affiliated Hospital of Shenzhen University, Number 3002, Sungang west road, Futian district, Shenzhen 518035, China; ^2^Department of spine surgery, The Shenzhen Second People's Hospital, Number 3002, Sungang west road, Futian district, Shenzhen 518035, China; ^3^Shenzhen Youth Spine Health Center, Shenzhen, Number 2008, Sungang west road, Futian district, 518000, China; ^4^Department of otolaryngology, The First Affiliated Hospital of Shenzhen University, Number 3002, Sungang west road, Futian district, Shenzhen 518035, China

## Abstract

**Objective:**

Adolescent idiopathic scoliosis (AIS) affects 1%-4% of adolescents in the early stages of puberty, but there is still no effective prediction method. This study aimed to establish a prediction model and validated the accuracy and efficacy of this model in predicting the occurrence of AIS.

**Methods:**

Data was collected from a population-based school scoliosis screening program for AIS in China. A sample of 884 children and adolescents with the radiological lateral Cobb angle ≥ 10° was classified as an AIS case, and 895 non-AIS subjects with a Cobb angle < 10° were randomly selected from the screening system. All selected subjects were screened by visual inspection of clinical signs, the Adam's forward-bending test (FBT), and the measurement of angle of trunk rotation (ATR). LR and receiver operating characteristic (ROC) curves were used to preliminarily screen the influential factors, and LR models with different adjusted weights were established to predict the occurrence of AIS.

**Results:**

Multivariate LR and ROC curves indicated that angle of thoracic rotation (adjusted odds ratios (AOR) = 5.18 − 10.06), angle of thoracolumbar rotation (AOR = 4.67 − 7.22), angle of lumbar rotation (AOR = 6.97 − 8.09), scapular tilt (area under the curve (AUC) = 0.77, 95% CI: 0.75-0.80), shoulder-height difference, lumbar concave, and pelvic tilt were the risk predictors for AIS. LR models with different adjusted weights (by AOR, AUC, and AOR+AUC) performed similarly in predicting the occurrence of AIS compared with multivariate LR. The sensitivity (82.55%-83.27%), specificity (82.59%-83.33%), Youden's index (0.65-0.67), positive predictive value (82.85%-83.58%), negative predictive value (82.29%-83.03%), and total accuracy (82.57%-83.30%) manifested that LR could accurately identify patients with AIS.

**Conclusions:**

LR model is a relatively high accurate and feasible method for predicting AIS. Increased performance of LR models using clinically relevant variables offers the potential to early identify high-risk groups of AIS.

## 1. Introduction

Adolescent idiopathic scoliosis (AIS) is defined as a three-dimensional (3D) structural deformity of the spine and is diagnosed on the basis of having a radiological lateral Cobb angle ≥ 10° [1], which occurs mainly in children and adolescents. AIS affects 1%-4% of adolescents in the early stages of puberty and is often neglected by teachers and parents, because it tends to be painless [2]. Although brace and surgery have been proven to be an effective intervention for AIS, it is believed that early detection of potential clinical signs related to scoliosis enables effective intervention on relatively small curves [3, 4]. Therefore, school scoliosis screening (SSS) has been applied to identify schoolchildren who may be at risk for scoliosis before their curvature progression.

In China, SSS mainly includes the following assessment methods: visual inspection, Adam's forward bending tests (FBT), and scoliometer measurements [5, 6]. However, the use of SSS remains controversial, mainly due to the unnecessary radiography caused by overreferral, which is related to the low positive predictive value (PPV) of SSS [7]. Previous studies reported that in identifying Cobb angle more than 20° the PPV varied from 17.4%-43.6%, and for requiring treatment it varied from 5.0%-9.4% [8, 9]. To improve the PPV of scoliosis screening, it is necessary to screen predicting indicators and establish the precise prediction model to accurately identify AIS patients.

A previous meta-analysis study showed that combined use of multiple clinical signs evaluated by examiners may increase the PPV to detect scoliosis during SSS [10]. Incorrect posture (e.g., shoulder imbalance, thoracic kyphosis, and scapular tilt) refers to an abnormal body state in which the individual's body cannot maintain a standing stability and normal function of tissues and organs in an upright body posture [11]. Evidence showed that children and adolescents with certain signs of incorrect posture may be associated with the progress to scoliosis, and these abnormal features may be helpful to predict the occurrence of AIS [12, 13]. To our knowledge, most of the previous research mainly focused on the prediction of curve progression in AIS patients or using a complex analysis method that was difficult for clinicians to understand [14–16]. Nault et al. built a prediction model using general linear methods with the 3D spine parameters and clinical parameters as predictors and found a PPV of 79% to identify a curve of 35° [15]. Xu et al. developed a genetic predictive model to evaluate the discriminative power between AIS patients and normal controls found a remarkably higher proportion of risk score in patients than in the controls (59.0% vs. 28.9%) [16]. However, it is still unclear whether the signs of incorrect posture commonly used in SSS can effectively predict the occurrence of AIS. Logistic regression (LR) model which is widely used to distinguish binary variables has been shown to have a high diagnostic accuracy in many diseases, but there are still few relevant studies to explore its predictive effects on the occurrence of AIS.

Therefore, we collected data from the 2019 School Scoliosis Screening Program for AIS (SSSPA) in China. We assumed that some signs of incorrect posture may be associated with the occurrence of AIS, and the combined use of multiple predictors could improve the accuracy of the prediction model. We aimed to examine the prevalence of incorrect posture stratified by AIS and to establish a prediction model basing on LR method with different adjusted weights, so as to improve the prediction accuracy and provide targeted prevention strategies for AIS.

## 2. Methods

### 2.1. Subjects and Data Collection

Data of the study was collected from the 2019 SSSPA in China, which is an ongoing school scoliosis screening program targeted for Chinese children and adolescents (1st-12th grade). SSSPA, as part of the national public health project, which is conducted and administered by the Shenzhen Youth Spine Health Center (SYSHC) of the Shenzhen Second People's Hospital with a national scoliosis screening standardized protocol (GB/T 16133-2014) [17], collects large-scale population-based scoliosis-related data every year since 2013. Students in primary schools, junior high schools, and senior high schools were invited to participate in the screening program voluntarily. School scoliosis screening was performed by an experienced team of trained rehabilitation therapists from SYSHC using the visual inspection, Adam's FBT, and measurement of the angle of trunk rotation (ATR) using the scoliometer [7]. When students had an ATR > 5° or showed significant clinical signs of scoliosis would be referred for a radiography lateral Cobb angle to accurately measure the curvature of the spine [18].

To protect the privacy of the students, all subjects were screened for scoliosis in a closed room or tent and administered by research assistants without the presence of teachers or other school personnel (to avoid potential information bias). All data were collected from September 2019 to January 2020.

For the purpose of our study, subjects with a clinical diagnosis of congenital scoliosis or neuromuscular scoliosis would be excluded. In total, a sample of 884 students was classified as AIS patients (case group) with a Cobb angle ≥ 10°, and a random sample of 895 students with a Cobb angle < 10° (control group) was selected from the screening system.

### 2.2. Ethical Statement

This study was conducted in accordance with the Declaration of Helsinki and was approved by the Shenzhen Municipal Health Commission Institutional Review Board (ethics number: SWJGW201934). Written or oral informed consent was obtained from the parent or legal guardian of each participating student under 18 years old or from each participating student who were at least 18 years old.

### 2.3. School Scoliosis Screening

Students would be required to wear tight clothing and underwear before school scoliosis screening. All students who volunteered to participate in the scoliosis screening would be divided into two groups according to their gender, and each group of students entered one by one in a sealed tent or room to protect personal privacy. To improve the accuracy of body posture measurement, students would only wear underwear during the screening; if someone refused this request for certain reason (such as unwilling to let the examiners to see their bodies), we would respect their personal choice and allow them to wear tight clothing for screening. During the screening, the subjects would wear their shoes and maintain a natural standing posture, the distance between their feet would be required to be as wide as the shoulders, their eyes needed to look straight ahead, and the arms should sag naturally.

### 2.4. Measurements

Based on the previous evidence [10], the combined use of multiple clinical signs may improve the PPV to detect AIS. In our previous large-scale population-based (595,057 students) study, we found that some signs of incorrect posture could help us early detect the occurrence of AIS [19]. To explore the potential predictors and establish an accurate prediction model of the AIS, the measurement variables used in the study contained information from demographic information and multiple signs of incorrect posture [5].

**Figure 1:**
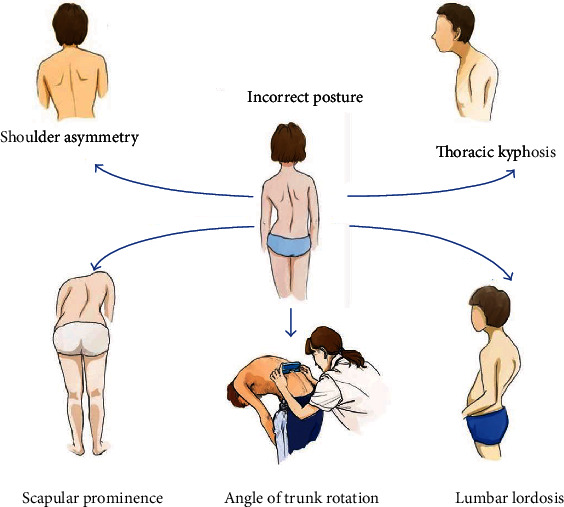
Measurement of incorrect posture.

Demographic characteristics included gender (boys or girls), age (year), and school category (primary school, junior high school, or senior high school). Incorrect posture was assessed by the visual inspection, Adam's FBT, and ATR (Figure 1). Each student participating in the screening was judged by two independent therapists separately. If the results were inconsistent, a third therapist would make a final judgment to minimize subjective bias. The standard visual inspection was performed in the upright position, and the examiners checked for spine alignment, shoulder asymmetry (e.g., shoulder-height difference), scapular prominence (e.g., scapular tilt), hip and pelvic obliquity (e.g., pelvic tilt), thoracic curvature (e.g., flat back, thoracic kyphosis), lumbar curvature (e.g., lumbar concave, lumbar kyphosis), distance of hands from the flanks, and length of the lower limbs and scapular [6]. The Adam's FBT was performed with the student's feet placed together, knees straight, while bending at the hips to nearly 90° with their arms freely hanging forward, palms together. Students with any significant physical signs were recorded. The ATR was measured with a scoliometer to quantitative assessment of the angle of thoracic rotation, angel of lumbar rotation, and angle of thoracolumbar rotation. When students were assessed with an ATR > 5° or with 1 or more significant physical signs of scoliosis, they would be rescreened by specially trained physicians and referred for a standing posteroanterior and lateral radiograph of the whole spine for final diagnoses [7], and those with a Cobb angle ≥ 10° measured by two independent experienced observers would be confirmed as AIS.

### 2.5. Statistical Analysis

First, descriptive analyses were conducted to describe the demographic characteristics and incorrect posture of children and adolescents stratified by AIS, chi-square (*χ*2) test or *t*-test was used to compare the differences between groups. Second, logistic regression (LR) models were applied to preliminarily screen the influential factors of AIS. Univariate LR models were performed to explore the association between incorrect posture and AIS, and multivariate LR was conducted to test the independent effects of each influential factors. Odds ratios (OR), adjusted odds ratios (AOR), and 95% confidence intervals (CI) were obtained from the LR models. Third, receiver operating characteristic (ROC) curves and corresponding area under the curve (AUC) scores were used to compare discrimination effects between different influential factors. Fourth, the total sample was randomly divided into a training data and a testing data according to a ratio of 7 : 3. The training data was used to build the prediction model, and the test data was used to evaluate the prediction effect. Demographic characteristics (e.g., gender and age) and incorrect posture (e.g., shoulder-height difference, scapular tilt, and ATR) as the predictors, and whether Cobb angle ≥ 10°as the dependent variable. LR models with different adjusted weights (by AOR, AUC, and AOR+AUC) were used to establish the prediction model in predicting the occurrence of AIS, and to compare the specific model statistics (sensitivity (Se), specificity (Sp), Youden's index (YI), positive predictive values (PPV), negative predictive values (NPV), and total accuracy (Ac)) with multivariate LR. All statistical analyses were conducted using IBM SPSS version 24.0 (IBM Corp, Armonk, NY, USA) and GraphPad Prism version 8.0 (GraphPad Software, San Diego, USA). A two-tailed *P* value of less than 0.05 was considered statistically significant.

## 3. Results

### 3.1. Demographic Characteristics of Children and Adolescents Stratified by AIS

As shown in Table 1, of the total sample analyzed, 884 (49.7%) students were diagnosed with AIS, and 895 (50.3%) students were non-AIS. AIS was more common in girls than in boys (64.8% vs. 36.1%, *χ*2 = 146.84, *P* < 0.001), and the girls-to-boys ratio was 1.84 : 1 in AIS group. The mean (standard deviation) age of the AIS group was higher than non-AIS group (13.14 ± 1.87 vs. 12.67 ± 1.96, *t* = −4.63, *P* < 0.001), and more than half of the AIS patients came from high school (*P* < 0.001).

### 3.2. Prevalence of Incorrect Posture Stratified by AIS

As shown in Table 1, except for lumbar kyphosis, the prevalence of all other incorrect postures was different between AIS group and non-AIS group. The angle of thoracic rotation was significantly greater in AIS group than in non-AIS group (rotate to the left: 10.2% vs. 2.0%; rotate to the right: 25.9% vs. 2.7%; *χ*2 = 272.54, *P* < 0.001). Compared with non-AIS group, AIS group had a higher angle of lumbar rotation than non-AIS group (rotate to the left: 31.3% vs. 6.8%; rotate to the right: 10.9% vs. 2.0%; *χ*2 = 261.45, *P* < 0.001).

### 3.3. Association between Influential Factors and AIS

As shown in Table 2, the univariate LR model (model 1) was used to identify the influential factors. Gender, age, shoulder-height difference, scapular tilt, lumbar concave, pelvic tilt, thoracic kyphosis, angle of thoracic rotation, angle of thoracolumbar rotation, and angle of lumbar rotation were significantly associated with AIS (*P* < 0.001).

Furthermore, a multivariate LR method (model 2) was applied to examine the independent effects of the influential factors associated with AIS. Table 2 showed that gender (AOR = 1.88), age (AOR = 1.09), shoulder-height difference (AOR = 2.98 − 4.17), scapular tilt (AOR = 2.23 − 2.53), lumbar concave (AOR = 2.61 − 2.67), pelvic tilt (AOR = 0.43), angle of thoracic rotation (AOR = 5.18 − 10.06), angle of thoracolumbar rotation (AOR = 4.67 − 7.22), and angle of lumbar rotation (AOR = 6.97 − 8.09) remained significantly associated with AIS (*P* < 0.05).

### 3.4. Compare the Discrimination Effects of Influential Factors Based on ROC Curves Analyses

**Figure 2:**
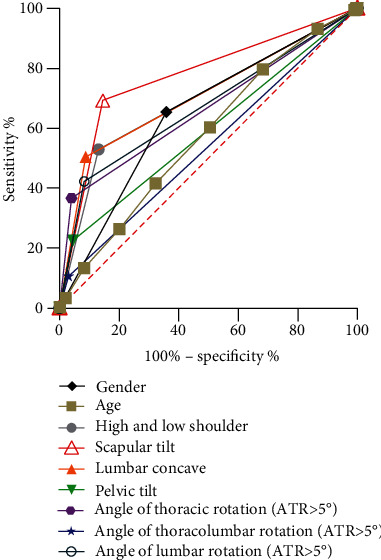
Results of ROC curve analysis by different risk factors. Gender: AUC = 0.65, 95% CI: 0.62-0.68, *P* < 0.001; age: AUC = 0.57, 95% CI: 0.54-0.61, *P* < 0.001. Shoulder-height difference: AUC = 0.70, 95% CI: 0.67-0.73, *P* < 0.001; scapular tilt: AUC = 0.77, 95% CI: 0.75-0.80, *P* < 0.001; lumbar concave: AUC = 0.71, 95% CI: 0.68-0.74, *P* < 0.001; pelvic tilt: AUC = 0.59, 95% CI: 0.56-0.62, *P* < 0.001; angle of thoracic rotation (ATR>5°): AUC = 0.66, 95% CI: 0.63-0.69, *P* < 0.001; angle of thoracolumbar rotation (ATR>5°): AUC = 0.54, 95% CI: 0.51-0.57, *P* = 0.023; angle of lumbar rotation (ATR>5°): AUC = 0.67, 95% CI: 0.64-0.70, *P* < 0.001. ROC curve results for flat back, thoracic kyphosis, and lumbar kyphosis were not shown because there was no statistical difference in its AUC value.

As shown in Figure 2, ROC curves and AUC scores were used to compare the discrimination effects between different influential factors. Similar to the results of LR models, gender (AUC = 0.65), age (AUC = 0.57), shoulder-height difference (AUC = 0.70), scapular tilt (AUC = 0.77), lumbar concave (AUC = 0.71), pelvic tilt (AUC = 0.59), angle of thoracic rotation (AUC = 0.66), angle of thoracolumbar rotation (AUC = 0.54), and angle of lumbar rotation (AUC = 0.67) could significantly distinguish AIS students from non-AIS students.

### 3.5. Prediction Model for AIS Based on LR Models with Different Adjusted Weights

As shown in Table 3, LR models with different adjusted weights (by AOR, AUC, and AOR+AUC) were used to establish the prediction model in predicting the occurrence of AIS and were compared with multivariate LR model in terms of their predictive effects. The final results indicated that compared to the multivariate LR model (Se = 82.55%, Sp = 82.59%, YI = 0.65, PPV = 82.85%, NPV = 82.29%, total Ac = 82.57%), LR models with adjusted weights by AOR, AUC, or AOR+AUC performed similarly in predicting the occurrence of AIS.

The mathematical equations of the prediction models were shown as follows:

Equation 1 = −7.124 + 0.705 X_1_ + 0.108 X_2_ + 0.705 X_3_ + 0.618 X_4_ + 0.790 X_5_ − 0.042 X_6_ − 0.965 X_7_ + 0.093 X_8_ − 2.235 X_9_ + 0.916 X_10_ + 1.406 X_11_ + 1.471 X_12_

Equation 2 = −3.648 + 0.367 X_1_ + 0.091 X_2_ + 0.316 X_3_ + 0.376 X_4_ + 0.435 X_5_ + 0.105 X_6_ − 0.984 X_7_ − 0.037 X_8_ − 2.122 X_9_ + 0.267 X_10_ + 0.275 X_11_ + 0.273 X_12_

Equation 3 = −3.787 + 0.448 X_1_ + 0.069 X_2_ + 0.650 X_3_ + 0.495 X_4_ + 0.756 X_5_ − 0.275 X_6_ − 0.827 X_7_ − 0.241 X_8_ − 1.955 X_9_ + 1.001 X_10_ + 1.420 X_11_ + 1.201 X_12_

Equation 4 = −3.648 + 0.222 X_1_ + 0.058 X_2_ + 0.186 X_3_ + 0.213 X_4_ + 0.2546 X_5_ + 0.066 X_6_ − 0.984 X_7_ − 0.037 X_8_ − 2.122 X_9_ + 0.713 X_10_ + 0.166 X_11_ + 0.163 X_12_

Abbreviations: X_1_: gender; X_2_: age; X_3_: shoulder-height difference; X_4_: scapular tilt; X_5_: lumbar concave; X_6_: pelvic tilt; X_7_: flat back; X_8_: thoracic kyphosis; X_9_: lumbar kyphosis; X_10_: angle of thoracic rotation; X_11_: angle of thoracolumbar rotation; X_12_: angle of lumbar rotation.

Equation 1 was the multivariate logistic regression model.

Equation 2 was the multivariate logistic regression model adjusted by weighting AOR value.

Equation 3 was the multivariate logistic regression model adjusted by weighting AUC value.

Equation 4 was the multivariate logistic regression model adjusted by weighting AOR and AUC value.

## 4. Discussion

In the 2019 SSSPA, students with scoliosis could be identified by the visual inspection of clinical signs and the Adam's FBT. Moreover, the application of scoliometer to measure the angle of trunk would help to improve the accuracy of screening. Our study was consistent with a previous systematic review conducted by Dunn et al. for the US Preventive Services Task Force (USPSTF) which suggested that AIS could be identified with Adam's FBT, scoliometer, or both [20]. Although estimation of predictive value and sensitivity was variable in different countries or population, our study conducted in the Chinese children and adolescents showed that the LR models could be trained to detect the occurrence of AIS and to identify cases with a curve ≥10°, with higher values than previous scoliosis screening in sensitivity, specificity, Youden's index, PPV, NPV, and total accuracy. Therefore, our LR models could be considered as a high accurate and feasible method in improving PPV and avoid unnecessary radiograph examination for scoliosis screening.

The univariate LR and ROC curves results indicated that the incorrect posture was associated with AIS among children and adolescents. After adjusted for covariates, multivariate LR model showed that angle of trunk may have the strongest relationship with AIS. Our results were consistent with a prospective 2-year follow-up study which indicated that the maximum angle of trunk was related to the severity of AIS compared to healthy adolescents [21]. An investigation study conducted in 27 AIS patients also showed that 3D trunk shape measured by angle of thoracic at each vertebral level was highly correlated with radiological deformity [22]. Consistent with the previous research [23], we found that shoulder-height difference, scapular tilt, lumbar concave, pelvic tilt, and other clinical signs were significantly associated with the occurrence of AIS. Using biomechanics and three-dimensional spatial positioning methods, some researcher speculated the alterations of shoulder, scapular, and lumbar spine could be considered as the adaptive compensation or muscle activation strategies in AIS patients [24–26]. Interestingly, pelvic tilt to the left (AOR = 0.43, 95% CI: 0.23-0.81) was considered as a protect indicators in our study. The reason may be that most scoliosis occurs on the right side, so the pelvis is prone to tilt to the left in order to maintain the balance of the body posture [27].

Furthermore, the prediction model based on LR models with different adjusted weights (by AOR, AUC, and AOR+AUC) in the current study indicated that LR has its own merit on predicting the occurrence of AIS. THE multivariate LR model has the advantages to filter the mess influencing factors easily. The adjusted odds ratio value of each factor could be compared directly and was easy to give the professional interpretation. In addition, we established prediction models based on the LR method with different adjusted weights; the combined use of AOR and AUC could merge their respective advantages and showed a high prediction accuracy. AOR aimed to reflect the relationship between the independent variable and dependent variable, and AUC was suitable for evaluating the diagnostic performance of the indicators. Interestingly, there were no significant differences between the multivariate LR model and LR models with different adjusted weights in the prediction accuracy. The possible reason was related to the relatively small sample size of cases and the outcome variable being a binary variable. Increasing sample size and multiclassification of outcomes (e.g., Cobb angle < 10°, 10° ≤ Cobb angle < 20°, 20° ≤ Cobb angle < 40°, and 40° < Cobb angle) were needed in the further study, to verify the predictive performance of different LR models.

Several aspects may contribute to the superiority of our prediction model. First, using only data from visual inspections of clinical signs and angle of the trunk, our LR models showed a higher and comparable prediction accuracy than the previous research. In the study by Karachalios et al. [28], the PPV ranged from 4.8%-13.3%; Fong et al. [10] and Yawn et al. [8] reported an improved PPV from 29.3% to 81.0% in their study. Our LR models showed the PPV ranged from 82.85% to 83.58% (sensitivity of 82.55%-83.27%, specificity of 82.59%-83.33%, total accuracy of 82.57%-83.30%) when AIS is identified with a Cobb angle ≥ 10°, so the prediction models established by LR methods might be a feasible and effective method to reduce the high false-positive rate of school scoliosis screening. Second, our prediction model used only several visual inspections of clinical signs and angle of the trunk and showed a high prediction accuracy in diagnosing AIS with a Cobb angle ≥ 10°, and no additional data were required. Yang et al. developed a deep learning algorithm for scoliosis screening and reported a PPV of 85.2%-89.4% (sensitivity of 80.7%-84.0% and specificity of 58.0%-90.0%) when identifying scoliosis cases with a Cobb angle ≥ 20° by using unclothed back images [29]. Compared with their results, our prediction models achieved a similar diagnosis accuracy but using only the traditional statistical methods (logistic regression and ROC curve), and the modeling was simpler and medically interpretable (AOR and AUC). The training and verification of the prediction models just used the screening data (the visual inspections of clinical signs and ATR) without additional data collection. Moreover, our study used a Cobb angle > 10° as a cut-off value, which may help us early identify the mild AIS patients and provided targeted interventions to slow or stop the progress of scoliosis.

The present study had several limitations that were worth noting. First, due to the cross-sectional nature of the data, it was not possible to make causal inferences. Second, our data mainly came from subjective physical examinations; although the measurement results in the study were assessed by two independent observers, measurement bias between observers for the severity of incorrect posture might exist. Third, although gender, age, and incorrect posture were showed to be important factors for the occurrence of AIS, other relevant influencing factors (e.g., genetics, hormone, and nutritional status) [30–32] were not investigated in our study.

## 5. Conclusion

Our prediction models based on LR method can be potentially applied in routine scoliosis screening without unnecessary radiation exposure and offer a relatively high accurate and feasible method for incorporating clinical signs to predict the occurrence of AIS among children and adolescents. Increased performance of LR models using clinically relevant variables offers the potential to early identify suspicious AIS patients and provide early warning for timely intervention and treatment of these high-risk groups. Our findings showed that angle of trunk rotation >5° could be considered as the best predictor to identify the occurrence of AIS, when the combined use of ATR > 5° and that the appearance of clinical signs of incorrect posture such as shoulder-height difference, scapular tilt, and lumbar concave would help to accurately predict the occurrence of AIS in the school scoliosis screening. However, due to the limitations of cross-sectional research data, more large-scale longitudinal studies are needed in the future to verify the external validity and robustness of the LR prediction model.

## Figures and Tables

**Table 1:** Demographic characteristics of participants and the prevalence of incorrect posture stratified by AIS (*N* = 1,779).

Variables	Non-AIS group (Cobb < 10°) n (%)	AIS group (Cobb ≥ 10°) *n* (%)	*χ*2/*t*	*P* value
Total	895 (50.3)	884 (49.7)		
Gender			146.84	<0.001
Boys	572 (63.9)	311 (35.2)		
Girls	323 (36.1)	573 (64.8)		
Age (year)^a^	12.67 ± 1.96	13.14 ± 1.87	-4.63	<0.001
School category			34.29	<0.001
Primary school	359 (40.1)	239 (27.0)		
Junior high school	374 (41.8)	458 (51.8)		
Senior high school	162 (18.1)	187 (21.2)		
Shoulder-height difference			333.96	<0.001
Normal	779 (87.0)	410 (46.4)		
Left shoulder height	73 (8.2)	252 (28.5)		
Right shoulder height	43 (4.8)	222 (25.1)		
Scapular tilt			554.97	<0.001
Normal	764 (85.4)	268 (30.3)		
Tilt to the left	85 (9.5)	349 (39.5)		
Tilt to the right	46 (5.1)	267 (30.2)		
Lumbar concave			346.90	<0.001
Normal	814 (90.9)	450 (50.9)		
Left concave	31 (3.5)	181 (20.5)		
Right concave	50 (5.6)	253 (28.6)		
Pelvic tilt			130.22	<0.001
Normal	860 (96.1)	694 (78.5)		
Tilt to the left	23 (2.6)	68 (7.7)		
Tilt to the right	12 (1.3)	122 (13.8)		
Flat back			4.57	0.033
Normal	893 (99.8)	875 (99.0)		
Abnormal	2 (0.2)	9 (1.0)		
Thoracic kyphosis			14.35	<0.001
Normal	886 (99.0)	851 (96.3)		
Abnormal	9 (1.0)	33 (3.7)		
Lumbar kyphosis			0.16	0.694^b^
Normal	891 (99.6)	882 (99.8)		
Abnormal	4 (0.4)	2 (0.2)		
Angle of thoracic rotation			272.54	<0.001
Normal (ATR: 0-5°)	853 (95.3)	565 (63.9)		
Rotate to the left (ATR > 5°)	18 (2.0)	90 (10.2)		
Rotate to the right (ATR > 5°)	24 (2.7)	229 (25.9)		
Angle of thoracolumbar rotation			41.82	<0.001
Normal (ATR: 0-5°)	872 (97.4)	796 (90.0)		
Rotate to the left (ATR > 5°)	9 (1.0)	42 (4.8)		
Rotate to the right (ATR > 5°)	14 (1.6)	46 (5.2)		
Angle of lumbar rotation			261.45	<0.001
Normal (ATR: 0-5°)	816 (91.2)	511 (57.8)		
Rotate to the left (ATR > 5°)	61 (6.8)	277 (31.3)		
Rotate to the right (ATR > 5°)	18 (2.0)	96 (10.9)		

Abbreviations: AIS: adolescent idiopathic scoliosis; n: number; ATR: angle of trunk rotation. ^a^Age were presented as the mean (standard deviation). ^b^Using chi-square test continuity correction calculation.

**Table 2:** Association between potential risk factors and AIS among Chinese children and adolescents (*N* = 1,779).

Variables	AIS: model 1^a^			AIS: model 2^b^		
	OR	95% CI	*P*	AOR	95% CI	*P*
Gender						
Boys	1.00			1.00		
Girls	3.26	2.69-3.96	<0.001	1.88	1.43-2.48	<0.001
Age (1-year increase)	1.14	1.08-1.19	<0.001	1.09	1.02-1.17	0.016
Shoulder-height difference						
Normal	1.00			1.00		
Left shoulder height	6.56	4.92-8.74	<0.001	2.98	1.88-4.72	<0.001
Right shoulder height	9.81	6.93-13.89	<0.001	4.17	2.49-7.01	<0.001
Scapular tilt						
Normal	1.00			1.00		
Tilt to the left	11.71	8.89-15.41	<0.001	2.23	1.43-3.46	<0.001
Tilt to the right	16.55	11.75-23.30	<0.001	2.53	1.53-4.16	<0.001
Lumbar concave						
Normal	1.00			1.00		
Left concave	10.56	7.09-15.72	<0.001	2.61	1.55-4.40	<0.001
Right concave	9.15	6.62-12.66	<0.001	2.67	1.77-4.03	<0.001
Pelvic tilt						
Normal	1.00			1.00		
Tilt to the left	3.66	2.26-5.94	<0.001	0.43	0.23-0.81	0.009
Tilt to the right	12.60	6.91-22.99	<0.001	1.83	0.91-3.71	0.093
Flat back						
Normal	1.00			1.00		
Abnormal	4.59	0.99-21.32	0.052	1.39	0.25-7.89	0.707
Thoracic kyphosis						
Normal	1.00			1.00		
Abnormal	3.82	1.82-8.03	<0.001	1.48	0.62-3.57	0.381
Lumbar kyphosis						
Normal	1.00			1.00		
Abnormal	0.51	0.09-2.77	0.431	0.32	0.04-2.59	0.282
Angle of thoracic rotation						
Normal (ATR: 0-5°)	1.00			1.00		
Rotate to the left (ATR > 5°)	7.55	4.50-12.66	<0.001	5.18	2.85-9.44	<0.001
Rotate to the right (ATR > 5°)	14.41	9.34-22.23	<0.001	10.06	6.11-16.56	<0.001
Angle of thoracolumbar rotation						
Normal (ATR: 0-5°)	1.00			1.00		
Rotate to the left (ATR > 5°)	5.11	2.47-10.57	<0.001	7.22	3.18-16.38	<0.001
Rotate to the right (ATR > 5°)	3.60	1.96-6.60	<0.001	4.67	2.28-9.55	<0.001
Angle of lumbar rotation						
Normal (ATR: 0-5°)	1.00			1.00		
Rotate to the left (ATR > 5°)	7.25	5.38-9.77	<0.001	6.97	4.84-10.05	<0.001
Rotate to the right (ATR > 5°)	8.52	5.09-14.26	<0.001	8.09	4.46-14.68	<0.001

Abbreviations: AIS: adolescent idiopathic scoliosis; OR: odds ratio; AOR: adjusted odds ratio; CI: confidence interval; ATR: angle of trunk rotation. ^a^Model 1 is a univariate logistic regression model. ^b^Model 2 is a multivariate logistic regression model.

**Table 3:** Comparison of prediction effects of different logistic regression models.

Indicator	Model 1^a^	Model 2^b^	Model 3^c^	Model 4^d^
Se	82.55%	83.27%	83.27%	83.27%
Sp	82.59%	82.59%	83.33%	82.59%
YI∗	0.65	0.66	0.67	0.66
PPV	82.85%	82.97%	83.58%	82.97%
NPV	82.29%	82.90%	83.03%	82.90%
Total Ac	82.57%	82.94%	83.30%	82.94%

Abbreviations: Se: sensitivity; Sp: specificity; YI: Youden's index; PPV: positive predictive value; NPV: negative predictive value; Ac: accuracy. ∗Calculated by Sensitivity+Specificity-1. ^a^Model 1 is multivariate logistic regression model. ^b^Model 2 is a multivariate logistic regression model adjusted by weighting AOR value. ^c^Model 3 is a multivariate logistic regression model adjusted by weighting AUC value. ^d^Model 4 is a multivariate logistic regression model adjusted by weighting AOR and AUC value.

## Data Availability

The questionnaire and datasets used and analyzed during this study are available from the corresponding authors upon reasonable request.
